# Tiny homes—big movement: building a permanent and affordable housing option for people with severe mental illness

**DOI:** 10.3389/fpubh.2025.1516751

**Published:** 2025-06-17

**Authors:** Amy Blank Wilson, Melissa L. Villodas, Thava Mahadevan, Emily Bosman, Jamie Swaine, John H. Gilmore, Lee Bowman, Alaina Money-Garman

**Affiliations:** ^1^School of Social Work, University of North Carolina at Chapel Hill, Chapel Hill, NC, United States; ^2^Department of Social Work, George Mason University, Fairfax, VA, United States; ^3^The Center for Excellence in Community Mental Health, Department of Psychiatry, University of North Carolina at Chapel Hill, Chapel Hill, NC, United States; ^4^XDS, Inc., Pittsboro, NC, United States; ^5^Garman Homes, Morrisville, NC, United States; ^6^Legion Company, Chapel Hill, NC, United States

**Keywords:** housing, community partnerships, severe mental illness, affordable housing, tiny homes, public mental health

## Abstract

**Background:**

Ensuring an adequate supply of affordable housing is one of the most pressing public health challenges facing the United States. This challenge is particularly pressing for people with severe mental illness living on incomes 25% below the federal poverty level, placing them at increased risk of housing insecurity.

**Description:**

This paper presents a community case study of the Tiny Homes Village (THV) demonstration project. In this project a community partnership used tiny homes to create a new affordable housing option for people with severe mental illness.

**Results:**

The THV built 15 tiny homes through a public/private cross-sector partnership consisting of a private non-profit organization, a university, a community mental health center, and construction companies. All 15 homes have the same floor plan and were constructed at the same time using a Blitz Build model in 90 business days at a cost of approximately $50,000 per home. Each home is built on a permanent foundation, and includes 416 square feet of interior, heated space and five living spaces: a full bathroom, a bedroom, an open-concept kitchen and living room, and a covered front porch that provides an additional 96 square feet of unheated space. The tiny homes are located within a village that offers several amenities and a range of community-based services. This community case study demonstrates the power of public-private partnerships to tackle some of our most complex and entrenched social problems while also providing a blueprint for how to expand the affordable housing options for people with severe mental illness.

## 1 Introduction

Having an affordable place to live is foundational to people’s health, mental health, and wellbeing and it decreases health care costs (1–3). Research has demonstrated that affordable housing is an important “health generating good” because it helps to ensure people can pay for resources such as food, medicine, and health care services (1). Additionally, research has also found that a lack of affordable housing (e.g., eviction, substandard housing, housing instability) creates emotional strains that can negatively impact mental health (4).

As many as a third of the people experiencing homelessness in the United States have a severe mental illness (3). Permanent Supportive Housing (PSH) is an evidence-based approach to supportive housing services in the mental health field that has been found to reduce homelessness, improve mental health outcomes and reduce healthcare costs among people with severe mental illness (3, 5), making it a valuable tool for promoting community integration. However, the effectiveness of PSH depends on the availability of existing affordable housing options in communities where clients live. While PSH offers individualized services that support community integration, this service cannot ensure that communities have an adequate supply of affordable housing options to meet demand. Instead, PSH engages strategies that support the individual client’s ability to compete successfully for a very limited supply of existing affordable housing stock in the private rental market. As a result, the potential effectiveness and scalability of PSH is constrained by the significant shortage of affordable housing units in the existing housing stock in the U.S.

The potential effectiveness of PSH is further limited by the financial gap that many people with severe mental illness experience each month related to housing costs. Specifically, many people with mental health conditions such as schizophrenia and other psychotic disorders, bipolar disorder, and major depression rely on government assistance programs such as Supplemental Security Income (SSI) for financial assistance. SSI provided $943 per month in financial support in 2024 (6)—an amount that falls 25% below the Federal Poverty Level. More importantly from a housing perspective, the amount of financial support people receive from SSI is not enough to pay monthly rents in most housing markets in the U.S. For example, the median cost for a one-bedroom apartment in the five most affordable housing markets in the United States is 72–88% ($680–$830) of this monthly income. While the median monthly rental cost for a one-bedroom apartment in the five most expensive rental markets in the United States are three to four and a half times higher than monthly SSI payments ($2,670–$4,300) (7). These examples illustrate the stark financial gap that many people with severe mental illness face each month related to housing and highlight the need to engage efforts focused on increasing the supply of affordable housing units available to people with severe mental illness living in community-based settings.

The services typically offered by PSH provide a critical foundation of support for people with severe mental illness living in community settings. PSH has advanced efforts to support people with severe mental illnesses’ access to existing housing units within rental markets using strategies such as providing housing vouchers, offering tenancy supports and building relationships with landlords. However, these individually focused service strategies alone cannot overcome the housing challenges created by the rising cost of housing, growing shortage of affordable housing units in the United States, and low quality of existing affordable housing, such as single room occupancy (SRO) hotels that have provided affordable housing options for people with severe mental illness for decades. This community case study supports efforts to expand the stock of affordable housing available to people with severe mental illness by presenting details of the Tiny Homes Village (THV) demonstration project. This demonstration project represents an innovative program that sought to use tiny homes to expand the continuum of permanent and affordable housing options available to people with severe mental illness. In this community case study, we provide an overview of the THV demonstration project’s goals, a description of the key partnerships that supported the development of tiny homes as a new permanent and affordable housing option, and a description of the stages of development of the new housing option. We also include a description of the design and construction of the tiny homes that highlight innovative aspects of the project, and a discussion of lessons learned during the construction of the THV to support future applications elsewhere.

## 2 Context

### 2.1 Project overview

The Tiny Homes Village (THV) is a demonstration project that uses tiny homes to create a new permanent and affordable housing option for people with severe and persistent mental illness (e.g., schizophrenia and other psychotic disorders and/or major affective disorders). The THV provides proof of concept that well-designed tiny homes can be used as an affordable and permanent housing option for people with severe mental illness. It includes 15 tiny homes, which were built within the context of a village that includes amenities such as a community clubhouse/wellness center, walking trails, an outdoor pavilion, and easy access to public transportation and a range of community-based services. The THV is also located adjacent to an alternative therapeutic environment, providing future residents with access to a number of additional services that focus on optimizing health and wellness, such as yoga, horticultural therapy, art and music classes, and cooking lessons. People with severe mental illness will be given priority consideration for living in the THV, which is expected to be operational by the summer of 2025. Future residents will receive permanent supportive housing services, including individualized mental health service plans as needed. Evaluation activities have been integrated into the development of the THV to support the scalability of the project through replication in other communities.

### 2.2 Key project partnerships

The Tiny Homes Village was built through a public-private cross-sector partnership. The key project partners that supported vertical construction of the tiny homes are outlined in Table 1. The THV public-private partnership was led by Cross Disability Services, Inc. (XDS)—a small community private nonprofit agency—and the School of Social Work (SSW) at the University of North Carolina at Chapel Hill (UNC-CH). As illustrated in Table 1, XDS is the project owner, developer, and operator of the THV, and the SSW at UNC-CH is the primary project sponsor. Together, these two organizations led efforts to develop and fund the project by supporting the development of a Design Team that included partners from social work, mental health treatment, construction and project management. The Center for Excellence in Community Mental Health, a large, university-affiliated community mental health agency, is another key project partner with the THV through their commitment to providing mental health services to residents once the homes are built. The THV Design Team worked together for 6 years to develop, fund, and build the THV. The Design Team also worked to broaden the community partnerships to include a larger team of professionals to support the development and construction of the tiny homes. This included two community partners that played a critical key role in the vertical construction of the tiny homes. Legion Company LLC, a privately owned construction company, is a key member of the design team through their work that focused on overseeing and facilitating construction activities throughout the project’s three construction phases: permitting, horizontal construction, and vertical construction. This included coordinating and overseeing permitting activities, contracting with construction professionals, facilitating the development of the master site plan and construction schedule, and communicating with construction professionals throughout all phases of the project. Garman Homes, a privately owned home building company, is also another key partner in vertical construction through their work to develop the final design for the tiny homes and to build all 15 homes.

**Table 1:** Key project partners.

Key project partners	Description	Overview of role(s) in the partnership
Cross Disability Services, Inc. (XDS)	Community-based private nonprofit agency	Project owner, developer, and operator of the Tiny Homes Village
School of Social Work (SSW), University of North Carolina at Chapel Hill (UNC-CH)	Public academic institution	Primary project sponsor
Center for Excellence in Community Mental Health (CECMH)	University-affiliated community mental health organization	Mental health service provider
Legion Company LLC	A private real estate strategy and advisory firm for private and public sectors	Construction project management
Garman Homes	Private homebuilding company	Home builder for vertical construction

### 2.3 Project funding

The THV has raised $2.3 million in grants and other funds to support the four stages of development and construction described below. The THV Design Team obtained funding through a variety of philanthropic sources, including grants and gifts from the university and a number of foundations and governmental and health care entities. Private donors, including individuals, community and faith-based organizations, and businesses, provided additional monetary and in-kind contributions. Table 2 provides details on project funders organized by the Project Phase described in more detail below.

**Table 2:** Project funders.

Funder	Phases			
	Conceptual	Permitting	Horizontal	Vertical
UNC-CH Center for Excellence in Community Mental Health	X	X	X	X
UNC-CH School of Social Work	X	X	X	X
Private donors	X	X	X	X
Oak Foundation		X	X	X
Goodman Award for Strategic Partnerships	X			
C. Felix Harvey Award from the University of North Carolina at Chapel Hill	X			
Cardinal Innovations Healthcare	X		X	
Wells Fargo			X	
Vaya Health			X	
Chatham County Housing Trust Fund			X	X
Alliance Health				X
A.J. Fletcher Foundation				X

### 2.4 Project phases

The development and construction of the tiny homes involved four phases: (1) conceptualization, (2) permitting, (3) horizontal construction, and (4) vertical construction. A brief overview of each project phase, which includes descriptions of key activities the Design Team engaged to support the successful progression of work on this project, is provided below.

#### 2.4.1 Phase 1: conceptualization (2015–2017)

The conceptualization phase focused on examining the feasibility and acceptability of using tiny homes as a new form of permanent and affordable housing for people with severe mental illness. Three key activities occurred during this phase. First, XDS developed the Tiny Home Collaborative with the support of the UNC Center for Excellence in Community Mental Health (CECMH). The Tiny Home Collaborative received the Goodman Award for Strategic Partnership (8) that provided seed money to support the development of a model tiny home. Second, the Tiny Home Collaborative worked in partnership with Habitat for Humanity of Chatham County, an organization committed to building affordable housing, to construct a model tiny home at the Chatham County Fair in the fall of 2015. Third, the Tiny Home Co-director and faculty at UNC-CH SSW received the C. Felix Harvey Award from the University of North Carolina at Chapel Hill. This grant supported evaluation efforts that used the model tiny home to elicit end-user feedback (i.e., input from service providers and people with severe mental illness) on how to design future tiny homes and their surrounding communities to meet the needs of people with severe mental illness. The results of this evaluation were published in 2022 and informed the design of the tiny homes presented here and the community where they are located (5).

#### 2.4.2 Phase 2: permitting (2018–2019)

The permitting phase focused on obtaining the approvals needed to begin construction of the tiny homes. Two key activities took place during this phase. First, the Design Team developed preliminary plans for the design of the tiny homes and a site plan for the village where they would be located. Second, the necessary entitlements (i.e., approvals) to begin construction activities were obtained. These entitlements included zoning approvals, which focused on the use type (e.g., office, residential, mixed) and density (i.e., number of residential units per area) for the THV, as well as preliminary sub-division approvals, which included approvals for the project’s construction plans from all relevant departments (i.e., county planning, environmental, and fire).

#### 2.4.3 Phase 3: horizontal construction (2020–2023)

Horizontal construction focused on preparing the THV site for the construction of the tiny homes and other community amenities. A key activity in this phase involved contracting with a construction company to clear and grade the land to prepare the homesites. Preparing the homesites for construction entailed widening the road leading to the village to ensure emergency vehicle access, creating an erosion control system, connecting the THV to the public water system, building a self-contained septic system, and connecting individual homesites to water and power prior to building the homes in vertical construction.

#### 2.4.4 Phase 4: vertical construction (January 2023–August 2023)

The vertical construction phase focused on constructing the tiny homes themselves. Key activities in this phase included finalizing the design of the tiny homes, building them, and obtaining certificates of occupancy for all 15 homes. A more detailed description of each of these activities is provided in Program Elements below.

## 3 Program elements

### 3.1 Tiny home design

**Figure 1:**
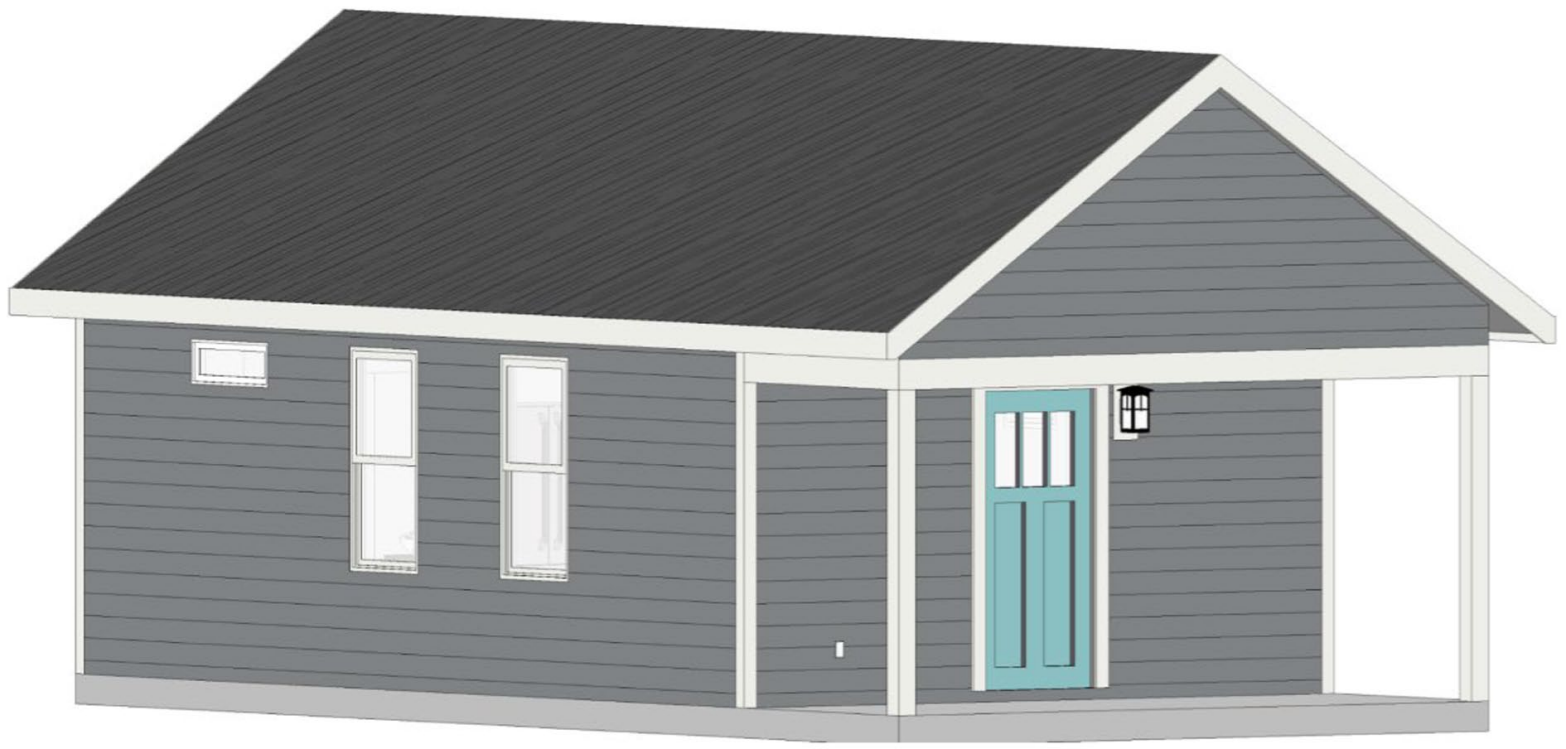
Tiny home architectural rendering.

**Figure 2:**
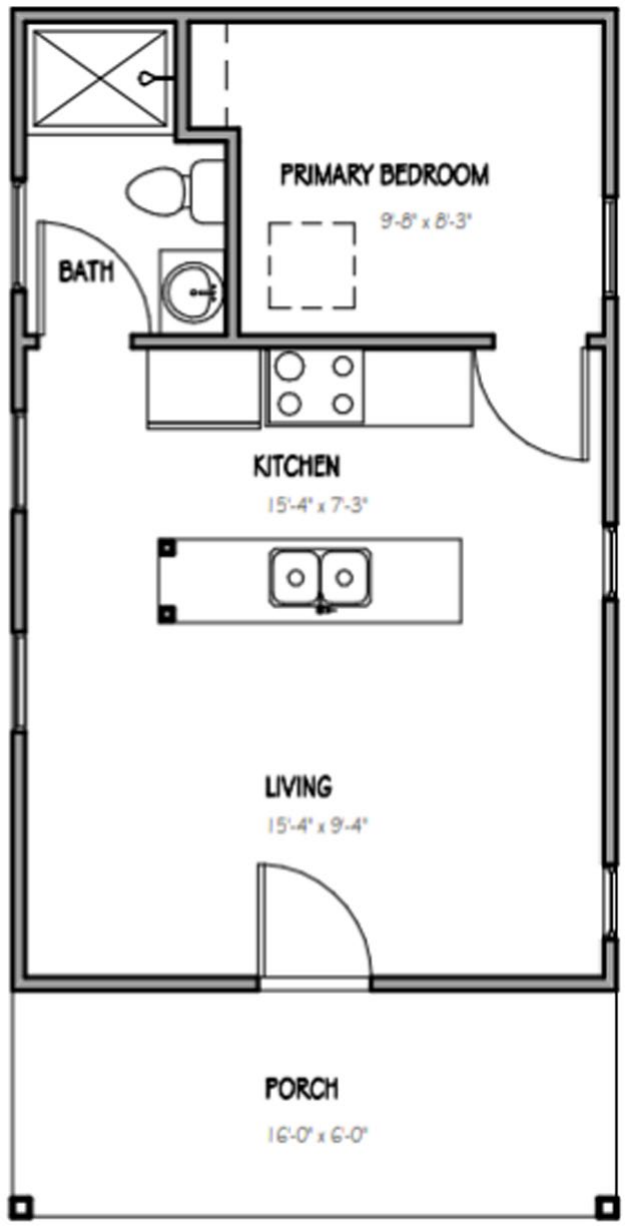
Tiny home floorplan.

The first innovative aspect of this project is the design and construction of 15 tiny homes, each of which is a stand-alone home that does not share walls with any other home, and are built on permanent foundations. Additionally, the design of the tiny homes incorporates feedback from people with severe mental illness and mental health service providers. Key elements of the design of the tiny home include the following information. All 15 homes are built on a permanent slab foundation made of concrete and include 416 square feet of interior heated space. Additionally, all 15 tiny homes use the same design (illustrated in Figure 1) and the same floor plan (illustrated in Figure 2). As the floor plan shows, each tiny home includes five living spaces located on a single floor. The living spaces include: a full bathroom (9′ × 5′), a bedroom (9′8″ × 8′3″), a kitchen (15′4″ × 7′3″), and a living room (15′4″ × 9′4″). Each home also has a covered front porch (16′ × 6′) that provides an additional 96 square feet of unheated space. The tiny home floor plan includes a kitchen and living room that engages an open concept design with the island serving as the anchor that joins the two spaces together. The bedroom is designed to have space for a full-size bed and open shelving built into the wall for storage. The bathroom includes a 3′ × 4′ walk-in shower (which is larger than the typical 3′ × 3′ size), a full-size toilet, and a vanity with faucet, sink, mirror, and storage space. Each tiny home has five windows, with at least one window in each interior living space and vinyl plank flooring throughout that is engineered to look like hardwood. Each home also has a shingle roof and the same color vinyl siding. The front door of each tiny home is painted one of five different colors to personalize the home.

### 3.2 Tiny home construction

The second innovative aspect of this project was how the homes were built. Specifically, in this project the home builder used an accelerated building schedule for the construction of the tiny homes that adapted the “Blitz Build” model developed by Habitat for Humanity (6) for use in this project. This accelerated building schedule involved building all 15 tiny homes at the same time over the course of 90 business days. Construction began on May 1, 2023 and was completed the week of August 28, 2023. Each home passed all local building authority inspections and received a certificate of occupancy within a week of construction being completed. Using the blitz building approach, all 15 homes were built simultaneously with all construction occurring on site. To accomplish this, a single construction schedule was developed wherein each step in the vertical construction process was executed at the same time for all 15 homes. Construction began with pouring the foundations for all the homes and continued in this manner throughout each step in the vertical construction process until the homes were completed. Based on feedback received during the project’s conceptualization phase, the plots of land for the homes were oriented to ensure that no two front porches faced one another. This was done to maximize the privacy of residents.

### 3.3 Tiny home cost

The third innovative aspect of our project was our ability to complete construction of the tiny homes below the original price point of $50,000 per home set in 2017. This price point was set using the median cost of construction per square foot for single-family homes in 2017, with the expectation that the homes would be similar in size to the model tiny home (326 square feet). The total construction cost for the 15 tiny homes, at 416 square feet of interior heated space and 96 square feet of exterior unheated space per home, was $690,773, with an average cost per home of $46,051. This included the cost of materials and labor associated with the vertical construction activities. Given this project’s structure, the cost of the land and the horizontal construction activities completed prior to vertical construction were considered separate and so were not included in the cost of the homes.

## 4 Discussion

This community case demonstrates how community based non-profit organizations can lead efforts to expand the affordable housing stock and provides a blueprint for how tiny homes can be used to expand the affordable and permanent housing options for people with severe mental illness. Over the course of this project, we learned several lessons that can inform future applications that are described below.

One of the earliest lessons we learned about building tiny homes affordably is that, when it comes to construction, smaller does not necessarily mean less expensive. We discovered this upon receiving preliminary cost estimates for the tiny homes that were well over $200 per square foot. While the reasons behind this are complex and beyond the scope of this project, it is important for community-based organizations to recognize that some construction costs, such as labor, do not depend on the size of the home and can actually be higher for projects that require specialized skills or services. During vertical construction, Garman Homes, our home building partner, used several strategies that helped counterbalance these challenges. First, they decided to build all 15 tiny homes at one time. This blitz build model of construction eliminated the redundancy in project overhead costs that would have occurred if the homes were built individually, while also optimizing the economy of scale of construction activities. Second, Garman Homes used a single design for all 15 homes, increasing the efficiency of construction and standardizing the costs of each home. Third, during the design process, our home building team extensively examined construction costs associated with the tiny home designs with the goal of optimizing both design and affordability. Fourth, rather than pricing the homes based solely on their square footage, the home builder used a new model, inviting trade partners to collaborate with them on an effort to expand affordable housing options that had both intrinsic and community value and to establish pricing accordingly.

The second lesson we learned about building tiny homes affordably is that it is possible to manage construction costs by managing the timing of construction activities. While construction professionals are well aware of the relationship between time and money in construction, they often operate under tight timelines. Since this demonstration project was not constrained by the deadlines present in many residential and commercial construction projects, we actively managed the timing of construction activities as a strategy to contain costs. As an example, we developed flexible timelines with construction partners, which managed costs by allowing our partners to fit work on our project into breaks or openings in their larger construction schedules. Additionally, we considered market conditions when making decisions about when and how to proceed with construction activities. This strategy led us to delay construction at times to control costs. For example, we waited to proceed with construction of the tiny homes until the impact of lumber shortages and other major supply chain disruptions that accompanied the global COVID-19 pandemic subsided.

The third lesson we learned in this project is the importance of including both interdisciplinary and cross-sector partners in our public/private partnership. These partnerships included disciplines as diverse as social work, mental health, and construction and project management, as well as professionals working in university, community nonprofit, and construction industry settings. We also learned that we could build strong partnerships when people were connected to and invested in our cause—in this case, building a new affordable and permanent housing option for people with severe mental illness.

We engaged several strategies that supported the success of our public-private partnership. First, we developed a core Design Team that took responsibility for the day-to-day operations of the project and met weekly to ensure progress and address problems as they arose. Additionally, each member of the Design Team worked to translate the project’s vision, goals, and progress to their respective organizations and constituents. This strategy helped build consensus and understanding within the larger organizations that were part of this community partnership. Additionally, one organization in this partnership, XDS, owned and operated the THV. This created a structure that streamlined the decision-making process as the project progressed across its stages of development.

## 5 Acknowledgement of conceptual or methodological constraints

While this demonstration project achieved its goal of using tiny homes to create a new permanent and affordable housing option for people with severe mental illness there are several constraints to note. First, this community case study presents the results of one community’s efforts to build a new form of affordable housing. The design decisions and construction reported in this community case study address the specific contextual and structural needs of this project. Further research is needed to determine the best way to use tiny homes to build affordable housing generally. Additionally, given that the focus of this project was on the construction of tiny homes, data related to outcomes for future residents is not available and should be addressed in future research. Additionally, the number of homes that could be built in this project was limited by federal regulations related to Medicaid, a key funding source for treatment services for people with severe mental illness. This constraint points to the need for organizations considering replication efforts and other future uses to carefully consider both internal and external factors when establishing the number of tiny homes they include in their community. It is also important to note that, our goal was to build homes that were both affordable and permanent, so we ensured that the construction of the tiny homes in this project satisfied all relevant building codes needed to obtain certificates of occupancy as single-family residences. We recognize that this approach to building tiny homes may differ from those employed in projects focused on using tiny homes to provide temporary shelter. This distinction points to an important goal of our project, which is to demonstrate the potential for tiny homes to be used as affordable and permanent housing. However, we stress that tiny homes are not a panacea that can solve the affordable housing crisis on their own. Rather, this project demonstrates that tiny homes are one option that can be added to the larger continuum of affordable housing stock. Additionally, the construction activities in this project were based on the site conditions where the homes were built. This included a number of infrastructure needs that may not be present in sites located within towns and cities that have more ready access to public infrastructure such as public water and sewers. Future projects will have to assess their own local conditions when building a site plan and considering how cost of living and infrastructure costs will shape affordability goals. Finally, in addition to the monetary donations described in the funding section of this paper, this project also received in-kind donations and discounts during vertical construction helped us achieve our goal of building the homes for less than $50,000 each.

## Data Availability

The original contributions presented in the study are included in the article/supplementary material, further inquiries can be directed to the corresponding author.

## Author’s note

Our project demonstrates the power of public-private partnerships to tackle some of our most complex and entrenched social problems.
